# Cost-effectiveness analysis of first-line treatment with crizotinib in ROS1-rearranged advanced non-small cell lung cancer (NSCLC) in Canada

**DOI:** 10.1186/s12885-021-08746-z

**Published:** 2021-10-29

**Authors:** Jaclyn M. Beca, Shaun Walsh, Kaiwan Raza, Stacey Hubay, Andrew Robinson, Elena Mow, James Keech, Kelvin K. W. Chan

**Affiliations:** 1 Ontario Health (Cancer Care Ontario), 525 University Ave, 3rd floor, Toronto, ON M5G 2L3 Canada; 2 Canadian Centre for Applied Research in Cancer Control, 525 University Ave, 3rd floor, Toronto, ON Canada; 3 Grand River Hospital, 835 King St W, Kitchener, ON Canada; 4 Kingston General Hospital, 76 Stuart St, Kingston, ON Canada; 5 Sunnybrook Odette Cancer Centre, Sunnybrook Health Science Centre, 2075 Bayview Ave TG 260, Toronto, ON Canada

**Keywords:** ROS1, Non-small cell lung cancer, Crizotinib, Cost-effectiveness

## Abstract

**Introduction:**

While no direct comparative data exist for crizotinib in ROS1+ non-small cell lung cancer (NSCLC), studies have suggested clinical benefit with this targeted agent. The objective of this study was to assess the cost-effectiveness of crizotinib compared to standard platinum-doublet chemotherapy for first-line treatment of ROS1+ advanced NSCLC.

**Methods:**

A Markov model was developed with a 10-year time horizon from the perspective of the Canadian publicly-funded health care system. Health states included progression-free survival (PFS), up to two further lines of therapy post-progression, palliation and death. Given a lack of comparative data and small study samples, crizotinib or chemotherapy studies with advanced ROS1+ NSCLC patients were identified and time-to-event data from digitized Kaplan-Meier curves were collected to pool PFS data. Costs of drugs, treatment administration, monitoring, adverse events and palliative care were included in 2018 Canadian dollars, with 1.5% discounting. An incremental cost-effectiveness ratio (ICER) was estimated probabilistically using 5000 simulations.

**Results:**

In the base-case probabilistic analysis, crizotinib produced additional 0.885 life-years and 0.772 quality-adjusted life-years (QALYs) at an incremental cost of $238,077, producing an ICER of $273,286/QALY gained. No simulations were found to be cost-effective at a willingness-to-pay threshold of $100,000/QALY gained. A scenario analysis assuming efficacy equivalent to the ALK+ NSCLC population showed a slightly more favorable cost-effectiveness profile for crizotinib.

**Conclusions:**

Available data appear to support superior activity of crizotinib compared to chemotherapy in ROS1+ advanced NSCLC. At the list price, crizotinib was not cost-effective at commonly accepted willingness-to-pay thresholds across a wide range of sensitivity analyses.

**Supplementary Information:**

The online version contains supplementary material available at 10.1186/s12885-021-08746-z.

## Introduction

Lung cancer is the most commonly diagnosed cancer in Canada and globally, accounting for 14% of the new cancer cases in both men and women, and one of the leading causes of cancer deaths with five-year relative survival rates for lung cancer patients in Canada of 14% for males and 20% for females [1]. Non-small cell lung cancer (NSCLC) is the most common type of lung cancer, accounting for 80 to 85% of all lung cancers [2].

ROS1 proto-oncogene tyrosine-protein kinase (ROS1) rearrangements are found in approximately 1–2% of NSCLC cases and are generally considered mutually exclusive from other oncogenic mutations commonly found in NSCLC e.g., epidermal growth factor receptor (EGFR), KRAS, or anaplastic lymphoma kinase (ALK) mutations [3]. ROS1 rearrangements are more commonly associated with adenocarcinomas, younger age, and light or never-smokers [3].

While many advances have been made in molecularly targeted treatment for NSCLC, there are limited treatment options specifically targeting ROS1+ NSCLC. However, ROS1+ gene-rearrangement has been demonstrated to be a predictive biomarker, as certain ALK inhibitors appear to also have ROS1 inhibitory activity [4]. Crizotinib is a tyrosine kinase inhibitor with demonstrated effectiveness against echinoderm microtubule-associated protein-like 4 (EML4)-ALK rearrangements as well as anti-tumour activity against biologically similar domains of ROS1 and another proto-oncogene receptor tyrosine kinase, MET [5, 6]. ROS1 and ALK receptor tyrosine kinases belong to the same insulin-receptor family, sharing close structural homology between the adenosine triphosphate (ATP)-binding kinase domains to which crizotinib binds with high affinity. The PROFILE 1001 trial was a multicentre, open-label, single-arm, phase I clinical trial that reported the efficacy and safety of crizotinib in a cohort of patients with ROS1+ NSCLC [7]. Originally designed with an initial dose-escalation phase, followed by an expansion phase to establish the recommended dose in enriched cohorts of patients predicted to have a clinical response based on molecular profiling, a protocol amendment permitted enrollment of an expansion cohort of patients with ROS1 rearrangements. At the data cut-off date of May 14, 2014, the primary endpoint, objective response rate (ORR) derived by investigator assessment, was 72% (95% CI: 58–84%) among patients with ROS1+ advanced NSCLC (*N* = 50), and 85.7% (95% CI: 42.1–99.6%) among the seven patients who had not received prior treatment. In the updated analysis, published in 2019 with a median 62.6 months of follow-up, the median progression-free survival (PFS) was 19.3 months (95% CI: 15.2–39.1) and the median OS was 51.4 months (95% CI: 29.3-not reached) [8].

In the absence of targeted therapy for ROS1+ NSCLC, treatment options used for NSCLC include traditional cytotoxic chemotherapy regimens (e.g., platinum-doublets, pemetrexed, docetaxel), and novel, more expensive checkpoint inhibitor immunotherapies (e.g., programmed cell death protein 1 (PD-1) inhibitor immunotherapies, nivolumab and pembrolizumab). The efficacy of these therapies in ROS1+ NSCLC patients is largely unknown due to a lack of evidence from clinical trial data. Although limited, the clinical effectiveness data available and biological rationale provide clinical interest to use crizotinib for treatment of ROS1+ NSCLC.

Cost-effectiveness analyses can provide further information for reimbursement decision-making by examining the comparative costs and benefits compared to existing options. While traditional chemotherapy agents are relatively inexpensive and some (e.g., platinum agents) may be used for a fixed number of treatment cycles, novel targeted drug therapies like crizotinib can cost nearly $8000 (CDN) a month and may be used as long as the patient continues to benefit (remains progression-free) [9]. Given the relatively higher cost of novel targeted agents compared to standard chemotherapy, [9] along with the uncertainty in comparative effectiveness data, economic evaluation decision modelling provides valuable synthesis of information to guide decision-makers about treatment choice, resource allocation and value for money. The objective of this study was to assess the cost-effectiveness of crizotinib compared to standard pemetrexed-based platinum-doublet chemotherapy for first-line treatment of ROS1+ advanced NSCLC, to inform recommendations for public drug reimbursement in Canada.

## Methods

### Model overview

The analysis included cost-effectiveness and cost-utility analysis of crizotinib compared with pemetrexed-based platinum-doublet chemotherapy for patients with previously untreated, ROS1+ advanced NSCLC. The outcomes included total costs, life-years (LYs), quality-adjusted life-years (QALYs), and incremental cost-effectiveness ratios (ICERs) per LY gained and per QALY gained. LYs measure the average expected survival time with each strategy, and the difference between strategies represent incremental LYs gained with crizotinib. QALYs incorporate effects on both quality (morbidity) and quantity (mortality) of life [10]. To calculate QALYs, LYs are multiplied by a quality of life weighting that measure preference for a given health state, known as health utility [10]. The ICER is a ratio of the difference in expected costs divided by difference in expected outcomes (either LY or QALYs), representing the additional cost associated with each additional (quality-adjusted) year of life gained with the crizotinib strategy A compared to usual care strategy B [10].

$$ ICER=\frac{Cost_A-{Cost}_B\ }{Effects_A-{Effects}_B}=\frac{\Delta  C}{\Delta  E} $$

The model was developed from the perspective of the publicly-funded Canadian health care system over a 10 year time horizon using a one-week model cycle length. Future costs and outcomes were discounted at a rate of 1.5% per year as per Canadian guidelines [11].

**Fig. 1:**
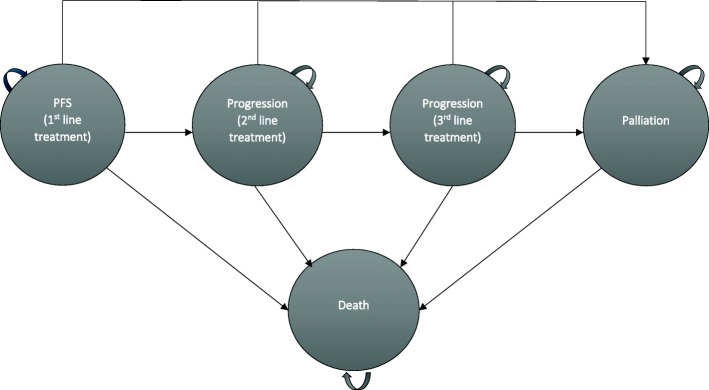
Model structure for Markov model PFS = progression-free survival

The cost-effectiveness model was developed in Microsoft Excel® (version 16.0) using a decision tree for ROS1 testing and a five-health state Markov model for the disease, which included health states for PFS, two post-progression survival states for further lines of therapy, palliation and death (Fig. 1).

Molecular testing was conducted using immunohistochemistry (IHC) followed by confirmatory fluorescence in situ hybridization (FISH) of positive staining results for the ROS1 mutation, based on test characteristics and frequency of ROS1+ (estimated to be 1.64% of NSCLC) [12, 13]. Patients with positive test results enter the Markov model in the PFS state and receive first-line therapy with crizotinib or pemetrexed-based platinum-doublet chemotherapy. Clinicians have suggested that a subset (estimated to be 50%) of patients receiving platinum-doublet chemotherapy may go on to receive pemetrexed maintenance, based on patients’ fitness and willingness to continue therapy. Patients who progress after receiving initial treatment with crizotinib who are eligible for further therapy are assumed receive treatment with standard of care options, starting with platinum-doublet chemotherapy and option of pemetrexed maintenance for second-line treatment. Patients initially treated with platinum-doublet chemotherapy receive second-line treatment with either a checkpoint inhibitor or docetaxel. Clinical input has suggested there is little evidence that these therapies differ in efficacy. Upon progressing, patients who received platinum-doublet chemotherapy as second-line treatment were assumed to receive either checkpoint inhibitor or docetaxel for their third-line treatment whereas patients who received either checkpoint inhibitor or docetaxel for their second-line treatment received the alternative for third-line treatment. Patients deemed unfit for subsequent treatment or who progressed after third-line treatment receive palliation until death. Patients could progress to death from any health state in the model.

### Clinical inputs

#### Progression-free survival

A literature review was conducted to identify relevant clinical evidence on efficacy and safety of crizotinib or pemetrexed-based platinum-doublet chemotherapy regimens for the treatment of ROS1+ advanced NSCLC (Additional file 1: Fig. 1). Given the absence of any randomized controlled trials or prospective trials for chemotherapy in patients with ROS1+ NSCLC, retrospective studies were also included. To estimate survival endpoints for the economic model, studies were excluded if they did not have Kaplan Meier (KM) time-to-event data for PFS. Seven studies (2 trials and 5 retrospective studies) were identified (Additional file 1: Table 1). Due to lack of comparative data, the small sample size of the studies, and the range of outcomes, there was no definitive source among the studies for robust efficacy data. Treatment effectiveness was estimated by pooling the time-to-event PFS data from all identified studies for crizotinib [7, 14, 15] and pemetrexed-based platinum-doublet chemotherapy [15–19]. PFS KM curves from each study were digitized using Engauge (version 4.1) software and individual patient data (IPD) were pooled (Additional file 1: Fig. 2). For studies with very small samples, each event and censoring time was accurately replicated from the plot. For studies with larger samples, patient-level IPD were approximated using the methods of Guyot et al [20].

**Fig. 2:**
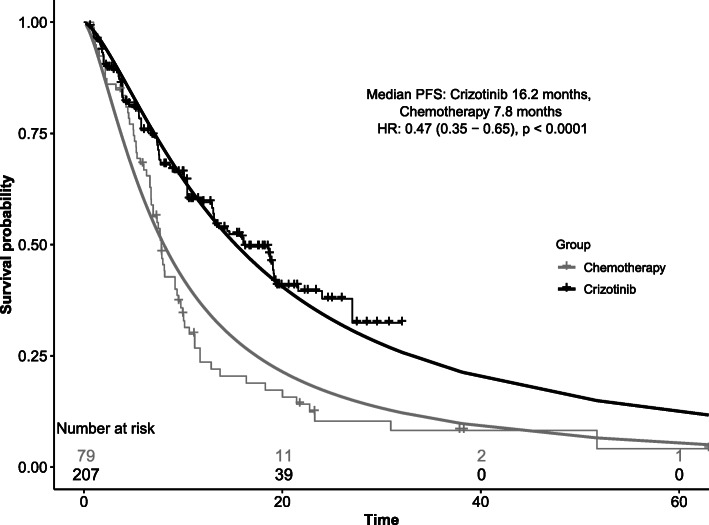
Kaplan-Meier curve and fitted log-logistic parametric curves from combined analysis for progression-free survival (PFS) among ROS1+ NSCLC patients

After pooling PFS data, parametric curves were fitted assuming the following distributions: exponential, Weibull, log-logistic, log-normal, generalized gamma and gompertz. Statistical tests failed to reject the assumption of proportional hazards (*p* > 0.05), suggesting it was reasonable to model with a common treatment parameter (proportional hazards or acceleration factor). The best fitting distribution (log-logistic) was chosen based on statistical information criteria, visual inspection of the curve and clinical plausibility [21] (Fig. 2). The survival functions were used to estimate monthly transition probabilities from the initial PFS state.

Scenario analyses for the clinical inputs included alternative data sources and approaches to modelling PFS. These include survival models estimated without using a common treatment parameter (wherein parametric curves were fitted to each treatment group separately), as well as using survival data from individual studies rather than the pooled analysis. Additionally, based on a review of previous health technology assessment (HTA) reviews, where it was noted that ROS1+ and ALK+ NSCLC share similar characteristics, [22] we conducted scenario analysis using PFS data from the phase III clinical trial for previously untreated ALK+ NSCLC, PROFILE 1014, [5] for comparability (Additional file 1: Fig. 3). The IPD for the ALK+ NSCLC data were recreated using the same methods as described above, and as the proportionality was deemed to be violated (*p* < 0.05), parametric curves were fitted individually to each treatment arm. In all scenario analyses, parametric survival distributions were fit using the same best practice approach considering statistical criteria, visual inspection and plausibility [21].

For patients who began treatment on chemotherapy, some may receive single-agent pemetrexed maintenance. While maintenance use was not reported in two of the studies contributing to the chemotherapy arm, it was assumed that use of maintenance would have an added PFS benefit. This was considered conservative because it would further improve PFS for the chemotherapy arm beyond what was observed in the pooled retrospective studies (and therefore reduce the incremental difference in PFS between treatment arms). Based on the PARAMOUNT trial, [23] which compared patients who receive pemetrexed maintenance relative to placebo after initial treatment with platinum-doublet chemotherapy, a hazard ratio (HR) of 0.62 (95% CI: 0.49–0.79) was applied to estimate the transition probabilities for patients continuing on pemetrexed maintenance (50% of patients remaining progression-free after induction) relative to patients undergoing no maintenance (Additional file 1: Fig. 4).

Patients in each arm progressed to second-line treatment, palliation, or death based on the time-varying transition probabilities from PFS, with 70% of patients assumed to receive second-line treatment, and remaining patients assumed to discontinue further therapy and receive palliative treatment (10%) or succumb to their disease (20%).

#### Progressed disease

Progression following all subsequent health states were estimated assuming exponential distributions. The risk of progression during second-line platinum-doublet chemotherapy was based on the median PFS value obtained from the combined analysis of chemotherapy studies, given this represented all available evidence for ROS1+ NSCLC. In the same manner as first-line, it was assumed that patients could undergo maintenance treatment (50%) and would experience an improvement in efficacy based on a HR of 0.62 [23]. Patients who received other second-line treatments were assumed to progress based on a weighted average of median PFS for checkpoint inhibitors or docetaxel (Additional file 1: Table 2), [24–27] with 60% assumed to receive third-line treatment, and the remaining equally likely to transition to palliation (20%) or death (20%). Efficacy was assumed to be similar for docetaxel or checkpoint inhibitors in both second- and third-line treatment. After third-line treatment, patients could either progress to palliation (60%) or to death (40%). The rate at which patients died from palliation was obtained from the TAX 317 study comparing patients receiving docetaxel or best supportive care [28].

### Cost inputs

Molecular test acquisition costs were used assuming upfront testing for ROS1 positivity to determine the total testing cost per case detected (Table 1). The test costs for IHC and FISH were obtained from the literature, [9] and adjusted to 2018 CAD costs using CPI health index [35].

**Table 1:** Input parameters

	Crizotinib	Chemotherapy	Source
MOLECULAR TESTING			
ROS1+ prevalence	1.64%		Rossi et al. [12]
Testing strategy proportion (IHC -> FISH)	100.0%		Assumption
IHC specificity rate	83.0%		Viola et al. [13]
IHC sensitivity rate	100.0%		Viola et al. [13]
FISH specificity rate	100.0%		Viola et al. [13]
FISH sensitivity rate	100.0%		Viola et al. [13]
EFFICACY PARAMETERS			
Treatment Efficacy			
First line PFS (Months)	16.2	7.79	Combined analysis (See text)
Median second-line PFS (Months) (Crizotinib arm – from combined analysis; Chemo arm – Checkpoint inhibitor/docetaxel)	7.79	3.26	Combined analysis (See text)
HR for pemetrexed maintenance	0.62		Paz-Ares et al. [23]
Median PFS for second- or third-line checkpoint inhibitor/docetaxel (Months)	3.26		Checkpoint inhibitor /docetaxel trials [24–27]
Median OS during palliation (Months)	4.60		Shepherd et al. [28]
*Percent receiving Treatments*			
% receiving pemetrexed maintenance	50.0%		Assumption
% receiving docetaxel versus checkpoint inhibitor	50.0%		Assumption
% receiving treatment during palliation	50.0%	0%	Assumption
*Transition splits*			
PFS to second-line treatment	70.0%		Assumption
Second-line treatment to third-line treatment	60.0%		Assumption
Third-line treatment to palliation	60.0%		Assumption
Proportion not treated after PFS to palliation (vs death)	33.3%		Assumption
Proportion not treated after second-line treatment to palliation (vs death)	50.0%		Assumption
COSTS			
Testing Costs			
IHC test cost	$42.36	NA	Djalalov et al. [9]
FISH test cost	$410.93	NA	Djalalov et al. [9]
Drug Costs			
Crizotinib (per tablet)	$130.00	NA	ODB Formulary [29]
Pemetrexed (per mg)	$0.21		CCO [30]
Docetaxel (per mg)	$0.27		CCO [30]
Pembrolizumab (per mg)	$44.00		CCO [30]
Nivolumab (per mg)	$19.56		CCO [30]
Other Costs			
Treatment administration + monitoring costs (monthly)	$1176.83	$1566.81	CCO, Schedule of benefits [31]
Total AE cost (first-line treatment)	$67.06	$318.68	OCC-CAT [32]
Palliation cost (monthly)	$3124.07		De Oliviera et al. [33]
UTILITY INPUTS			
Treatment-specific HRQoL (by line)			
PFS – first-line treatment	0.806	0.776	Solomon et al. [5]
Second- or third-line treatment	0.660		Shaw et al. [6]
Palliative Care	0.473		Nafees et al. [34]
*Disutility Estimates*			
Adverse event disutility estimate	−0.0194	− 0.0546	Calculation

*IHC* Immunohistochemistry, *FISH* Fluorescence in situ hybridization, *PFS* Progression Free Survival, *CCO* Cancer Care Ontario, *OCC-CAT* Ontario Case Costing – Costing Analysis Tool, *OS* Overall Survival, *AE* Adverse Event, *HRQoL* Health Related Quality of Life

For all regimens, an average patient body surface area (BSA) of 1.75m^2^ was assumed. Crizotinib costs were based on the current Ontario list price of $130.00 per 250 mg tablet, [29] given twice daily until progression or death [5–7, 14, 15]. Platinum-doublet chemotherapy consisted of a platinum agent (carboplatin or cisplatin) with pemetrexed, the preferred regimen of clinical experts. The cost for each treatment was obtained from Ontario’s cancer agency, Cancer Care Ontario (CCO), [30] and the dosing schedule obtained from clinical trials for a maximum of 6 cycles [5]. Carboplatin dosing was based on the target AUC according to the Calvert formula [36]. Costs for post-progression treatments with nivolumab, pembrolizumab and docetaxel were obtained from CCO, [5] and dosing and treatment schedules obtained from recent publications [23–27].

Administration costs, estimated from CCO costing data, included the costs of supplies, pharmacy, nursing, and administrative (clerical and management) staff for operation of the outpatient chemotherapy clinic. Monitoring costs were calculated using input from clinicians on resource utilization and the costs obtained from literature or the Physician and Laboratory Schedule of Benefits [31].

As no comparative adverse event (AE) data are available for the ROS1+ NSCLC population, comparative AE rates were obtained from the PROFILE 1014 study in ALK+ NSCLC for crizotinib and chemotherapy. Inpatient hospitalization and outpatient ambulatory care costs for diagnoses corresponding to grade III/IV AEs from the clinical trial were estimated from the Ontario Case Costing – Costing Analysis Tool [32].

The cost for palliation was obtained from a matched cohort study that estimated phase-specific net costs in Ontario, [33] and reported 12-month terminal care costs, which was used to calculate a weighted monthly cost of $3124.07.

In order to minimize bias in survival from differing number of lines of therapy available, three treatment lines were assumed in both groups followed by equal risks of death during palliation between groups. However, costs of a further line of therapy were included for some patients treated with crizotinib during the palliation state (50%), to avoid biasing the total costs in progressed states in favour of the crizotinib group.

### Utility inputs

Quality of life was not measured using any utility instruments in any of the included studies for ROS1+ NSCLC. Based on the similarity between ROS1+ and ALK+ NSCLCs, it was assumed that quality of life data from the PROFILE 1014 study in ALK+ NSCLC would be similar for patients with ROS1+ NSCLC. In the PROFILE 1014 trial, patients had similar quality of life at baseline, but patients on crizotinib experienced significant improvement during treatment compared to platinum-doublet chemotherapy, with mean EQ-5D index scores on treatment of 0.81 for crizotinib and of 0.72 for platinum-doublet chemotherapy. Similar to the approach taken by the National Institute for Health and Care Excellence (NICE) evidence review, we estimated a smaller utility difference between the groups, based on the difference in disutility due to AEs (0.034), to minimize the difference between the treatment strategies, resulting in a utility value of 0.77 for platinum-doublet chemotherapy (Table 1).

Patients who progressed on the first-line treatment were assigned a utility value of 0.66, obtained from previously treated ALK+ patients receiving docetaxel [6]. It was also assumed that treatment with checkpoint inhibitors and docetaxel were associated with similar quality of life in second- or third-line treatment. Utility values for palliative care were obtained from a valuation study with 100 participants administered a standard gamble interview about health states described by oncologists and nurses [34]. Scenario analyses were also conducted with equal utility scores between arms and with PROFILE 1014 values.

### Analysis

Probabilistic analysis was conducted with 5000 simulations to incorporate uncertainty in the model parameters together at once. Beta distributions were used for model parameters with values between 0 and 1 (probabilities, proportions and utilities), normal distribution for population values (e.g., BSA), and gamma distribution for costs. For the correlated uncertainty in the extrapolation parameters, we used normal distributions and the Cholesky decomposition. One-way sensitivity analyses on all input parameters of uncertainty and several scenario analyses were conducted to explore assumptions (Additional file 1: Table 3).

## Results

The combined recreated IPD for each study resulted in total sample sizes in crizotinib and chemotherapy arms of 207 and 79, respectively (Additional file 1: Fig. 2). This increased sample size from the variety of sources of heterogeneous populations provided a more robust dataset to model PFS using all available data for ROS1+ NSCLC. The estimated HR for crizotinib was 0.47 (95% CI: 0.35-0.65) in the pooled analysis, with median PFS of 16.2 months vs 7.8 months for chemotherapy.

**Fig. 3:**
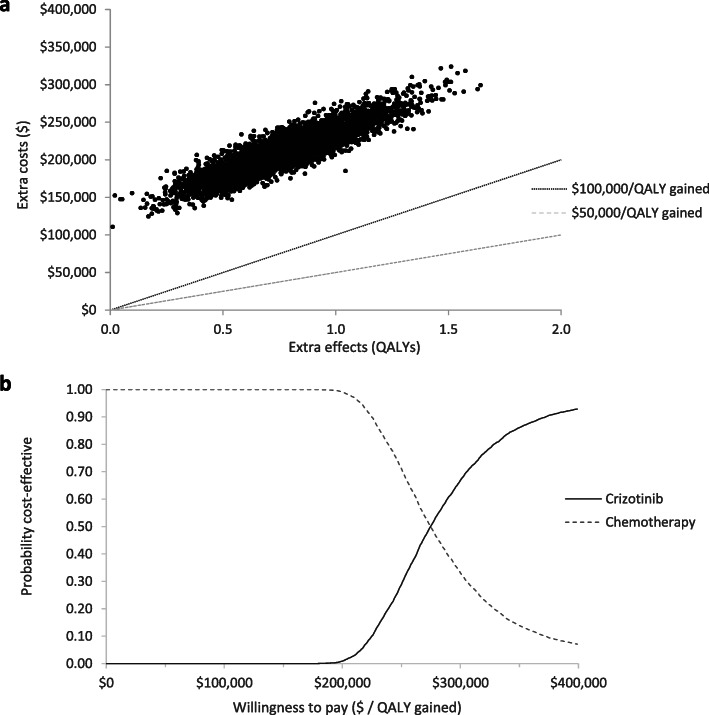
Results of 5000 simulations from probabilistic analysis a) on the cost-effectiveness plane and b) on a cost-effectiveness acceptability curve

In the base-case probabilistic analysis, crizotinib produced additional 0.885 LYs and 0.772 QALYs at an incremental cost of $238,077, producing an ICER of $273,286/QALY gained (Table 2). LY and QALY gains were produced at a higher cost in ~ 100% of probabilistic simulations, and none of the simulations produced results that were lower than a $100,000/QALY gained willingness-to-pay threshold (Fig. 3).

**Table 2:** Base-case results from the Markov model, based on 5000 simulations

	Crizotinib Arm	Chemotherapy Arm	Incremental
COSTS	$288,945	$78,071	$210,874
Testing	$7215	$0	$7215
PFS - First line	$246,127	$31,218	$214,909
Progressed - Second line	$11,879	$22,894	-$11,015
Progressed - Third line	$12,345	$13,959	-$1614
Palliation	$11,379	$10,001	$1379
LIFE YEARS (LYs)	3.349	2.465	0.885
PFS - First line	2.256	1.735	0.521
Progressed - Second line	0.680	0.288	0.392
Progressed - Third line	0.155	0.175	−0.020
Palliation	0.258	0.267	−0.009
QALYS	2.541	1.769	0.772
PFS - First line	1.819	1.337	0.482
Progressed - Second line	0.449	0.190	0.259
Progressed - Third line	0.102	0.115	−0.013
Palliation	0.170	0.126	0.044
ICER (Cost per LY gained)			$238,394
ICER (Cost per QALY gained)			$273,286

*ICER* Incremental cost effectiveness ratio, *PFS* Progression-free survival, *QALY* Quality adjusted life year

In the one-way deterministic sensitivity analysis, the model was most sensitive to assumptions about pemetrexed maintenance, in particular assumptions surrounding the percentage of patients in the comparator arm receiving pemetrexed maintenance as part of first-line treatment and the reduction in risk of progression (HR) due to maintenance (Additional file 1: Fig. 5). Since pemetrexed maintenance was associated with improved outcomes, assuming more patients received maintenance or that maintenance was more effective would improve the overall number of LYs and QALYs gained in the comparator arm at a relatively modest cost, thus increasing the ICER for crizotinib. The model was also sensitive to crizotinib drug costs, which represent a significant driver of overall costs in the treatment arm, and the risk of progression during second-line treatment in the crizotinib arm. When discounts of 30% are applied to the cost of crizotinib drug cost, crizotinib remains not cost effective at a willingness to pay threshold of $150,000/QALY gained.

Results of scenario analyses are shown in Table 3. A scenario based on the ALK+ population as proxy for ROS1+ NSCLC patients showed a slightly more favorable cost-effectiveness profile for crizotinib, as does a scenario assuming maintenance outcomes are captured in the ROS1+ chemotherapy data. Assuming no PFS or OS differences increases the ICER, as does assuming no quality of life difference between oral treatment with crizotinib and IV platinum-doublet chemotherapy.

**Table 3:** Outcomes for each scenario analysis

Scenario	Δ LYS	Δ QALYS	Δ COSTS	ICER (LY)	ICER (QALY)
Base case deterministic results	0.891	0.774	$210,203	$235,924	$271,418
1. ALK+ population as proxy for ROS1+ NSCLC patients This scenario will assume no difference between ALK+ and ROS1+ NSCLC patients and will utilize data from the PROFILE 1001 (Solomon 2014) study for PFS inputs.	1.075	0.897	$156,497	$145,642	$174,419
2. Second-best fitting curves for PFS The second-best fitting curves were selected in this scenario for PFS for the combined analysis in each arm.	1.014	0.867	$200,571	$197,752	$231,354
3. Exponential curves for PFS A constant risk was assumed based on an exponential distribution for PFS in this scenario for the combined analysis in each arm, to align with prior models for crizotinib.	1.131	0.952	$191,432	$169,269	$200,980
4. Individually fit curves Rather than modelling using a common treatment parameter, the individually fitted survival curves were selected for each arm for this scenario.	1.461	1.231	$242,217	$165,825	$196,732
5. PROFILE 1001 (Shaw 2014) alone Rather than using the combined analysis, the individually fitted curve from the PROFILE 1001 (Shaw 2014) study was used to inform efficacy inputs for the crizotinib arm.	1.242	1.063	$251,280	$202,360	$236,485
6. EUROS1 (Mazières 2015) data Rather than using the combined analysis for the crizotinib and chemotherapy arms, individually fitted curves from the Mazières 2015 study were used to inform efficacy inputs for each treatment arm.	0.891	0.759	$160,777	$180,447	$211,837
7. No PFS difference This scenario assumed no difference in PFS between the crizotinib and chemotherapy group. This is a conservative assumption as crizotinib appears to be associated with improved PFS outcomes. To achieve this the crizotinib PFS time-to-event values were substituted into the chemotherapy arm.	0.404	0.366	$121,524	$300,666	$331,738
8. No OS difference To deal with uncertainty surrounding the clinical benefit of crizotinib, this scenario assumed no difference in overall survival between the two groups.	0.000	0.175	$106,390	NA	$606,640
9. No added maintenance benefit Since uncertainty lies in whether the effect of maintenance is captured in the combined chemotherapy data, this scenario has removed the maintenance hazard ratio from both arms.	1.195	1.018	$216,744	$181,367	$212,829
10. Lower median PFS for second-line treatment (crizotinib arm) Since there is uncertainty regarding the median PFS estimate for patients undergoing platinum-doublet chemotherapy after progressing on crizotinib, a shorter median PFS value was used from Smit et al. [37]	0.594	0.578	$206,007	$346,825	$356,140
11. Equal first-line utility (crizotinib values) To test the uncertainty around utility value estimates, the utility value from the crizotinib arm was applied to both arms for first-line therapy.	0.891	0.714	$210,203	$235,924	$294,488
12. Equal first-line utility (chemotherapy values) To test the uncertainty around utility value estimates, the utility value from the chemotherapy arm was applied to both arms for first-line therapy.	0.891	0.696	$210,203	$235,924	$302,179
13. First-line utility values from ALK+ population To test the impact of using utility values from the PROFILE 1014 (Solomon 2014) trial for the chemotherapy arm (larger difference in utilities between groups)	0.891	0.864	$210,203	$235,924	$243,317
14. Proportion receiving active third-line therapy - 30% To test uncertainty surrounding proportion of patient’s receiving active third-line therapy, a lower value (30%) was applied to the model.	0.864	0.755	$209,033	$241,917	$276,986
15. Best estimate of 3 above parameters (10, 13, 14) – pCODR Reanalysis To test the impact of a combination of changes the above 3 parameter changes were applied together (Second line PFS of 4.2 months, ALK+ PROFILE 1014 (Solomon 2014) trial utilities and 30% receiving active third-line therapy).	0.584	0.659	$205,072	$351,140	$311,055

Δ = Difference, *ICER* Incremental cost-effectiveness ratio, *LY* Life year, *NSCLC* Non small-cell lung cancer, *OS* Overall survival, *pCODR* pan-Canadian Oncology Drug Review, *PFS* progression-free survival, *QALY* Quality-adjusted life year

## Discussion

This cost-effectiveness analysis estimated the total cost per course with administration and monitoring of first-line therapy with crizotinib for treatment of ROS1+ advanced NSCLC to be nearly $250,000, compared to the treatment cost of first-line platinum-doublet chemotherapy of approximately $31,000, resulting in an incremental cost of over $200,000 per patient. These high costs resulted from the high price of crizotinib as well as the potentially long duration of therapy of first-line targeted therapy used until progression; we estimated a median PFS for first-line therapy with crizotinib in ROS1+ NSCLC of over 16 months. Expensive checkpoint inhibitor immunotherapies, which also cost approximately $8000 per month, [38] can be used in later lines of therapy for NSCLC. However, not all patients are able to receive later lines of therapy and patients may not receive treatment for progressed disease for very long, as average duration of second and later lines of therapy from recent NSCLC trials was in the range of 2–4 months [24–27]. Thus, the use of the novel targeted first-line therapy for ROS1+ NSCLC appears to provide added clinical benefits but with considerable added costs, with the key driver being the cost of crizotinib, resulting in an ICER of $273,286/QALY gained. This economic analysis involved systematic use of all available clinical data in the relevant population and thorough exploration of uncertainty through a robust series of sensitivity and scenario analysis. We also analyzed a scenario using ALK+ advanced NSCLC as a proxy population for comparison with reviews conducted by HTA bodies in other countries. Given the high drug cost, crizotinib does not appear to be a cost-effective treatment option at list price in any scenario. The primary challenges in addressing the cost-effectiveness of crizotinib for ROS1+ NSCLC stem from the lack of comparative data, the small sample size of the studies, and range of observed outcomes for the given patient population. Given small sample sizes, we estimated effectiveness by pooling time-to-event data from all identified ROS1+ advanced NSCLC studies depicting KM curves for either treatment arm. Although alternative methods to pool survival data from individual studies exist, our approach is unique in that it estimated the time-to-event for patients through the meticulous digitization of KM data to estimate a KM curve for the pooled population. The resulting sample size for the combined analysis is significantly larger than the survival estimates from single studies.

At the time of this analysis, crizotinib had received positive funding recommendations for ROS1+ NSCLSC by several HTA bodies in other jurisdictions [22, 39–41]. However, none of these HTA bodies reviewed economic evidence derived from ROS1+ NSCLC, and instead were provided with cost-effectiveness evidence based on data from ALK+ NSCLC as proxy. The clinical behaviour of each appear similar to one another and possibly distinct from patients with NSCLC without driver mutations, [3, 15] most notably exhibiting similar objective response rates, (74% for ALK+ NSCLC [5] and 72% for ROS1+ NSCLC [7]). However, not all ALK-targeted agents have shown activity against ROS1+ NSCLC, [42] and it is often difficult to base reimbursement recommendations on evidence extrapolated from other disease settings. Thus, while some HTA bodies have made positive recommendations based on ALK+ NSCLC proxy data, we attempted to incorporate evidence directly from the ROS1+ advanced NSCLC population. Based on this evidence, the pan-Canadian Oncology Drug Review (pCODR), who makes funding recommendations to public drug plans for oncology drug products in Canada, issued a positive funding recommendation for crizotinib to treat ROS1+ NSCLC, conditional on pricing arrangements to improve the cost-effectiveness and budget impact of adoption [43]. Crizotinib has also been recommended for funding in Canada for ALK+ NSCLC, and for both ALK+ and ROS1+ NSCLC in other jurisdictions, but has not been found to be cost-effective at the list price in either setting. In our analysis, there was a very similar relative benefit of crizotinib in ALK+ and pooled ROS1+ NSCLC populations (HR = 0.48), but slightly longer absolute survival in ROS1+ NSCLC. Using ALK+ data in the current model, crizotinib was associated with 0.897 QALYs at an added cost of $156,497, resulting in a slightly lower ICER of $174,419/QALY gained. In the UK, using ALK+ NSCLC data as a proxy for ROS1+ NSCLC, NICE did not report the incremental benefits and costs separately, but reported an ICER around £50,000/ QALY gained, which is above their threshold for commonly-accepted willingness-to-pay [22]. In Canada, the pCODR estimated smaller added clinical benefit of crizotinib of between 0.131 and 0.211 QALYs for ALK+ NSCLC, at an additional cost between $36,548 and $37,387, resulting in an estimate ICER range of $173,570–$285,299 /QALY gained [44]. Other publications have found that among patients with ALK+ advanced NSCLC, first-line crizotinib provided 0.379 additional QALYs, cost an additional $95,043 compared with standard care, producing an ICER of $250,632/QALY gained, [9] and the major driver of cost effectiveness being drug price. While the estimates of benefit in our model using either ROS1+ or ALK+ NSCLC data were larger than other published estimates, our results were also associated with proportionally larger costs, leading to somewhat similar estimates of cost-effectiveness. Given the differences in approach between this model and previous models, which include the use of non-constant hazards of progression and longer time horizon of our model as data have evolved, these differences in model results are reasonable and still result in similar conclusions.

The primary limitation and main driver of the analysis was the uncertainty in comparative effectiveness. Though all data available at the time of the analysis were included, it is not possible to fully assess the comparability of the included studies. No IPD were available to adjust for any differences in baseline characteristics, and as information on the study populations were limited to the published reports, we could only superficially assess similarities in reported measures such as median age. We cannot determine the final characteristics of the pooled data for each treatment arm. Further, results were not stratified according to number of prior lines of therapy, as there was variation in the number of prior treatments patients received in the identified studies. However, it is notable that the crizotinib studies that included patients exposed to several prior lines of therapy still demonstrated impressive response rates and survival. We also included retrospective studies, which may involve patients who differ from those included in clinical trials. The aim of our analysis was to be inclusive by systematically combining all relevant clinical data for the given treatments in the population of interest. The inclusion of a variety of study populations may be preferred over relying upon a single study source, since it inherently incorporates heterogeneity across multiple study populations, and thus may better capture the overall population. However, naïve comparisons from different populations are always at risk of bias. In order to explore the uncertainty associated with our survival analysis, we conducted multiple scenario analyses, including the use of ALK+ as proxy for ROS1+ advanced NSCLC. The limited data also necessitated many additional assumptions, including assumptions that can have a big impact on the estimated survival benefits like effect of maintenance after platinum-doublet chemotherapy, as well as assumptions about comparative safety and health utility data for ROS1+ NSCLC patients, which were assumed to be generalizable from the proxy ALK+ NSCLC population. Pemetrexed maintenance and subsequent treatment data also were not from ROS1+ NSCLC populations. Although these approaches represent our best efforts to address the uncertainty, it does not replace high-quality, comparative clinical evidence in the population of interest. This study did not examine other potential sequencing scenarios, such as the use of crizotinib as second- or third line treatment, which future analysis may consider. Although the standard of care for non-squamous NSCLC has changed to incorporate immunotherapy, we did not feel this was appropriate for the first-line care of ROS1+ NSCLC population, given the uncertainty in effectiveness.

As targeted agents for rare driver mutations expands, and agents are approved without randomized phase III evidence, the need to try to do a comparative economic analysis with limited, early phase evidence is increasing. Available data appear to support superior activity of crizotinib compared to chemotherapy in ROS1+ advanced NSCLC. At the list price, crizotinib for ROS1+ NSCLC does not appear to be cost-effective at commonly accepted willingness-to pay thresholds across a wide range of sensitivity analyses. Given the potential clinical benefit for this rare subtype, price reductions may improve the value for money of this targeted treatment for ROS1+ NSCLC.

## Supplementary Information

**Additional file 1:** Additional information regarding the clinical data and model results. **Table 1.** Studies of crizotinib and chemotherapy for ROS1+ advanced NSCLC. **Table 2.** Summary of studies used to calculate risk of progression with second- and third-line docetaxel and checkpoint inhibitors. **Table 3.** List of scenario analyses. **Fig. 1.** PRISMA flow chart for ROS1 studies. **Fig. 2.** Kaplan-Meier curve from combined analysis for progression-free survival (PFS) among ROS1+ NSCLC patients. **Fig. 3.** Kaplan-Meier curve of PROFILE 1014 for progression-free survival (PFS) among ALK+ NSCLC patients for each treatment. **Fig. 4.** Kaplan-Meier curve and parametric curve of combined analysis for progression-free survival (PFS) for each treatment group with hazard ratio applied to patients undergoing maintenance pemetrexed in chemotherapy group. **Fig. 5.** Results from one-way deterministic sensitivity analysis.

## Data Availability

The datasets used and/or analysed during the current study available from the corresponding author on reasonable request.
