# Supplementary material for: Cost-effectiveness analysis of first-line treatment with crizotinib in ROS1-rearranged advanced non-small cell lung cancer (NSCLC) in Canada
Source: BMC Cancer. 2021 Oct 29;21:1162. doi: 10.1186/s12885-021-08746-z (PMC8556902; doi:10.1186/s12885-021-08746-z)
Supplement: Supplementary file 1 — Additional file 1: Additional information regarding the clinical data and model results. Table 1. Studies of crizotinib and chemotherapy for ROS1+ advanced NSCLC. Table 2. Summary of studies used to calculate risk of progression with second- and third-line docetaxel and checkpoint inhibitors. Table 3. List of scenario analyses. Fig. 1. PRISMA flow chart for ROS1 studies. Fig. 2. Kaplan-Meier curve from combined analysis for progression-free survival (PFS) among ROS1+ NSCLC patients. Fig. 3. Kaplan-Meier curve of PROFILE 1014 for progression-free survival (PFS) among ALK+ NSCLC patients for each treatment. Fig. 4. Kaplan-Meier curve and parametric curve of combined analysis for progression-free survival (PFS) for each treatment group with hazard ratio applied to patients undergoing maintenance pemetrexed in chemotherapy group. Fig. 5. Results from one-way deterministic sensitivity analysis. [file 12885_2021_8746_MOESM1_ESM.docx]

## Additional File 1: Additional information regarding the clinical data and model results

Cost-effectiveness analysis of first-line treatment with crizotinib in ROS1-rearranged advanced non-small cell lung cancer (NSCLC) in Canada

Authors: Jaclyn M Beca, MSc, Shaun Walsh, MSc, Kaiwan Raza, HBSc, Stacey Hubay, MD, FRCPC, Andrew Robinson, MD, FRCPC, Elena Mow, BSc (Pharm), ACPR, James Keech, MSc, Kelvin KW Chan, MD, FRCPC, MSc, PhD

Additional File 1 Contents:

Table 1. Studies of crizotinib and chemotherapy for ROS1+ advanced NSCLC

Table 2. Summary of studies used to calculate risk of progression with second- and third-line docetaxel and checkpoint inhibitors

Table 3. List of scenario analyses

Fig. 1. PRISMA flow chart for ROS1 studies

Fig. 2. Kaplan-Meier curve from combined analysis for progression-free survival (PFS) among ROS1+ NSCLC patients

Fig. 3. Kaplan-Meier curve of PROFILE 1014 for progression-free survival (PFS) among ALK+ NSCLC patients for each treatment

Fig. 4. Kaplan-Meier curve and parametric curve of combined analysis for progression-free survival (PFS) for each treatment group with hazard ratio applied to patients undergoing maintenance pemetrexed in chemotherapy group

Fig. 5. Results from one-way deterministic sensitivity analysis

References

Table 1. Studies of crizotinib and chemotherapy for ROS1+ advanced NSCLC

| Author | Treatment | Study Design | Population | Median PFS |
| --- | --- | --- | --- | --- |
| PROFILE 1001,  Shaw 2014  ^1^ | Crizotinib | A multicentre, open-label, single-arm, phase I clinical trial (n=50) | - Eligible patients ≥18 years had histologically confirmed, advanced NSCLC with a ROS1 rearrangement. - Median age: 53 years - ECOG performance status (PS) 0: 22 (44%), 1: 27(54%), 2: 1(2%) - Previous regimens for advanced disease 0: 7(14%), 1: 21(42%) and >1: 22 (44%) | 19.2 months |
| Wu 2018^2^ | Crizotinib | Single-arm, multicenter phase II clinical trial (n=127) | - Patients with ROS1+ NSCLC enrolled at 37 sites in China, Japan, South Korea, and Taiwan - Patients had received three or fewer lines of prior systemic therapies for advanced-stage disease or had one or more measurable tumor lesions with and ECOG PS of 0 or 1. - Median age: 51.5 (22.8-79.7) | 15.9 months |
| EUROS1, Mazieres 2015^3^ | Crizotinib | Retrospective study (n=30) | - Patients with FISH- confirmed ROS1+ NSCLC with stage IV disease treated with crizotinib - Crizotinib as a first- or second-line treatment (n=10, 32%), after two or more lines of chemotherapy (n=21, 68%) - Median age at diagnosis 50.5 years; age at treatment NR | 9.1 months |
| EUROS1, Mazieres 2015^3^ | Pemetrexed (single agent or platinum combination) | Retrospective study  (n=26) | - Patients with FISH- confirmed ROS1+ NSCLC with stage IV disease using chemotherapy before or after treatment with crizotinib - Use of maintenance NR | 7.2 months |
| Song 2016^4^ | First line palliative pemetrexed platinum combination | Retrospective study (n=12) | - Population from 2 sites in China between Jan 2010 and Dec 2014 - Among the 34 patients, 12 with advanced stage or recurrence were treated with pemetrexed-based first-line chemotherapy - Median age: NR - No maintenance used by any of the 12 patients | 6.8 months |
| Zhang 2016^5^ | Pemetrexed  (15 pts received crizotinib but all previously-treated) | Retrospective study  (n=28) | - East Asian population from a single centre in China between Oct 2013 and Feb 2016 - Median age: 57 years - ECOG PS: 0-1: 44 (93.6%) ≥2: 3 (6.4%) - Use of maintenance NR | 209 days / 6.9 months |
| Drilon 2016^6^ | First line pemetrexed platinum combination | Retrospective study (n=10) | - Stage IIIB/IV disease - Median age: 50 (18‐61) - 7/10 received maintenance pemetrexed | 23 months |
| Kim 2013^7^ | Pemetrexed | Retrospective Study (n=5) | - Population from Severance Hospital, Seoul, Korea between Jan 2005 and Feb 2012 - Median age: 55 (30-68) - Use of maintenance NR | Not reached |

ECOG PS = Eastern Cooperative Oncology Group performance status; FISH = Fluorescence in situ hybridization; n = number of patients; NR = not reported; NSCLC = non-small cell lung cancer; PFS = Progression-free survival

Table 2. Summary of studies used to calculate risk of progression with second- and third-line docetaxel and checkpoint inhibitors

| Treatment | Dose | N | Median PFS (months) | Source |
| --- | --- | --- | --- | --- |
| Nivolumab | 3 mg/kg every 2 weeks | 292 | 2.3 | Borghaei 2015^8^ |
| Nivolumab | 3 mg/kg every 2 weeks | 135 | 3.5 | Brahmer 2015^9^ |
| Pembrolizumab | 2 mg/kg every 3 weeks | 345 | 3.9 | Herbst 2015^10^ |
| Docetaxel | 75 mg/m^2^ of BSA every 3 weeks | 290 | 4.2 | Borghaei 2015^8^ |
| Docetaxel | 75 mg/m^2^ of BSA every 3 weeks | 137 | 2.8 | Brahmer 2015^9^ |
| Docetaxel | 75 mg/m^2^ of BSA every 3 weeks | 346 | 4.0 | Herbst 2015^10^ |
| Docetaxel | 75 mg/m^2^ of BSA every 3 weeks | 143 | 3.0 | Vansteenkiste 2015^11^ |

BSA = body surface area; N = number of patients; PFS = Progression-free survival

Table 3. List of scenario analyses

| Scenario | Base-Case Setting | Scenario Setting |
| --- | --- | --- |
| 1. ALK+ population as proxy for ROS1+ NSCLC patients   This scenario will assume no difference between ALK+ and ROS1+ NSCLC patients and will utilize data from the PROFILE 1014 (Solomon 2014)^12^ study for PFS inputs. | -PFS data: Combined Analysis  -Log-logistic distribution | - PFS data: PROFILE 1014  -Lognormal distribution |
| 1. Second best fitting curves   The second-best fitting curves were selected in this scenario for PFS for the combined analysis in each arm. | -Parametric distribution for combined analysis for crizotinib and chemotherapy PFS: Log-logistic | -Parametric distribution for combined analysis for crizotinib and chemotherapy PFS: Generalized gamma |
| 1. Exponential curves   A constant risk was assumed based on an exponential distribution for PFS in this scenario for the combined analysis in each arm, to align with prior models for crizotinib. | -Parametric distribution for combined analysis for crizotinib and chemotherapy PFS: Log-logistic | -Parametric distribution for combined analysis for crizotinib and chemotherapy PFS: Exponential |
| 1. Individually fit curves   Rather than modelling using a common treatment parameter, the individually fitted survival curves were selected for each arm for this scenario. | - PFS data for crizotinib and chemotherapy arm: Combined Analysis  -Log-logistic distribution | - PFS data for crizotinib and chemotherapy arm: Individually fitted data  -Log-logistic distribution |
| 1. PROFILE 1001 (Shaw 2014) alone   Rather than using the combined analysis, the individually fitted curve from the PROFILE 1001 (Shaw 2014)^1^ study was used to inform efficacy inputs for the crizotinib arm. | - PFS data for crizotinib arm: Combined Analysis  - Log-logistic distribution | - PFS data for crizotinib arm: PROFILE 1001  -Lognormal distribution |
| 1. EUROS1 (Mazières 2015) data   Rather than using the combined analysis for the crizotinib and chemotherapy arms, the individually fitted curves from the EUROS1 study (Mazières 2015)^3^ were used to inform efficacy inputs for each treatment arm. | - PFS data for crizotinib and chemotherapy arm: Combined Analysis | - PFS data for crizotinib and chemotherapy arm: EUROS1 |
| 1. No PFS difference   This scenario assumed no difference in PFS between the crizotinib and chemotherapy group. This is a conservative assumption as crizotinib appears to be associated with improved PFS outcomes. To achieve this the crizotinib PFS time-to-event values were substituted into the chemotherapy arm. | -Source of PFS data (crizotinib arm): Combined chemotherapy analysis  -PFS Maintenance HR: 0.62 | -Source of PFS data (crizotinib arm): Combined chemotherapy analysis  -PFS Maintenance HR: 1.00 |
| 1. No OS difference   To deal with uncertainty surrounding the clinical benefit of crizotinib, this scenario assumed no difference in overall survival between the two groups. | -Median OS (palliation): 4.60 | -(same PFS between two arms – see scenario 2)  -Median OS on BSC care (chemo arm): 12.05 months |
| 1. No added maintenance benefit   Since uncertainty lies in whether the effect of maintenance is captured in the combined chemotherapy data, this scenario has removed the maintenance hazard ratio from both arms. | -PFS maintenance (chemotherapy arm) & progression maintenance (crizotinib arm) HR: 0.62 | -PFS maintenance (chemotherapy arm) & progression maintenance (crizotinib arm) HR: 1.00 |
| 1. Lower median PFS for second-line treatment (crizotinib arm)   Since there is uncertainty regarding the median PFS estimate for patients undergoing platinum-doublet chemotherapy after progressing on crizotinib, a shorter median PFS value was used from Smit et al.^13^ | Median PFS for 2^nd^ line treatment (crizotinib arm): 7.79 months | Median PFS for 2^nd^ line treatment (crizotinib arm): 4.20 months |
| 1. Equal first-line utility (crizotinib values)   To test the uncertainty around utility value estimates, the utility value from the crizotinib arm was applied to both arms for first-line therapy. | Crizotinib arm: 0.81  Chemotherapy arm: 0.776 | Crizotinib arm: 0.81  Chemotherapy arm: 0.81 |
| 1. Equal first-line utility (chemotherapy values)   To test the uncertainty around utility value estimates, the utility value from the chemotherapy arm was applied to both arms for first-line therapy. | Crizotinib arm: 0.81  Chemotherapy arm: 0.776 | Crizotinib arm: 0.776  Chemotherapy arm: 0.776 |
| 1. First-line utility values from ALK+ population   To test the impact of using utility values from the PROFILE 1014 (Solomon 2014)^12^ trial for the chemotherapy arm (larger difference in utilities between groups) | Chemotherapy arm: 0.776 | Chemotherapy arm: 0.719 |
| 1. Proportion receiving active therapy in 3^rd^ line - 30%   To test uncertainty surrounding proportion of patient’s receiving active therapy in 3^rd^ line, 30% was applied to the model. | Proportion of patients who progress after 2^nd^ line and receive active 3^rd^ line therapy: 60% | Proportion of patients who progress after 2^nd^ line and receive active 3^rd^ line therapy: 30% |
| 1. Best estimate of 3 above parameters (10, 13, 14) – pCODR Reanalysis   To test the impact of a combination of changes the above 3 parameter changes were applied together (2^nd^ line PFS of 4.2 months, ALK+ PROFILE 1014 (Solomon 2014) utilities and 30% receiving active 3^rd^ line therapy | -Median PFS for 2L treatment (crizotinib arm): 7.79 months  -Chemotherapy arm: 0.776  -Proportion of patients who progress after 2^nd^ line and receive active 3^rd^ line therapy: 60% | -Median PFS for 2L treatment (crizotinib arm): 4.2 months  -Chemotherapy arm: 0.719  -Proportion of patients who progress after 2^nd^ line and receive active 3^rd^ line therapy: 30% |

HR = Hazard ratio; pCODR = pan-Canadian Oncology Drug Review; PFS = Progression-free survival; OS = Overall survival

| Database: Pubmed  Keywords: ROS1, lung cancer, prognosis  Search date: May 11, 2018  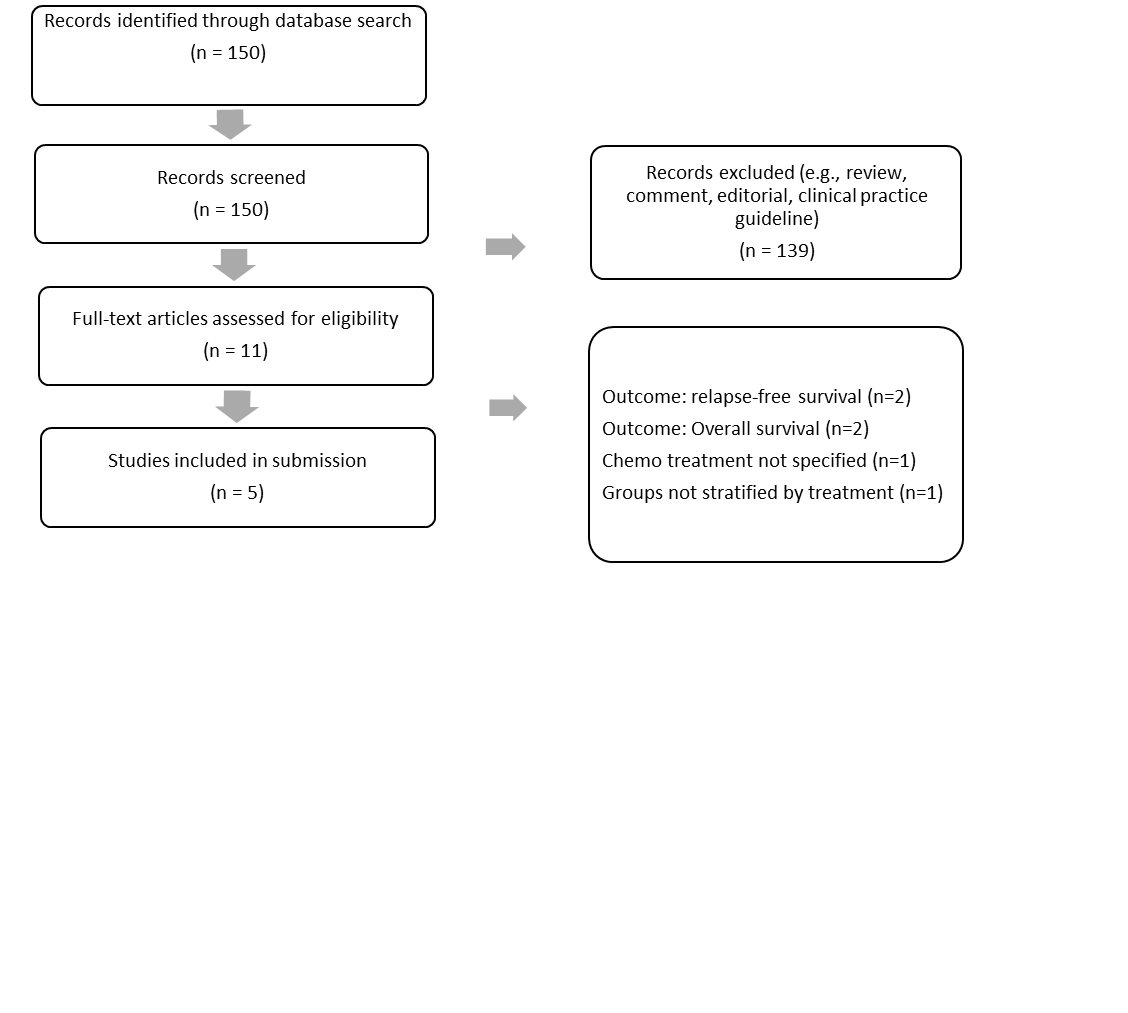 |
| --- |
| Database: Pubmed  Keywords: ROS1, lung cancer, crizotinib  Search date: May 14, 2018  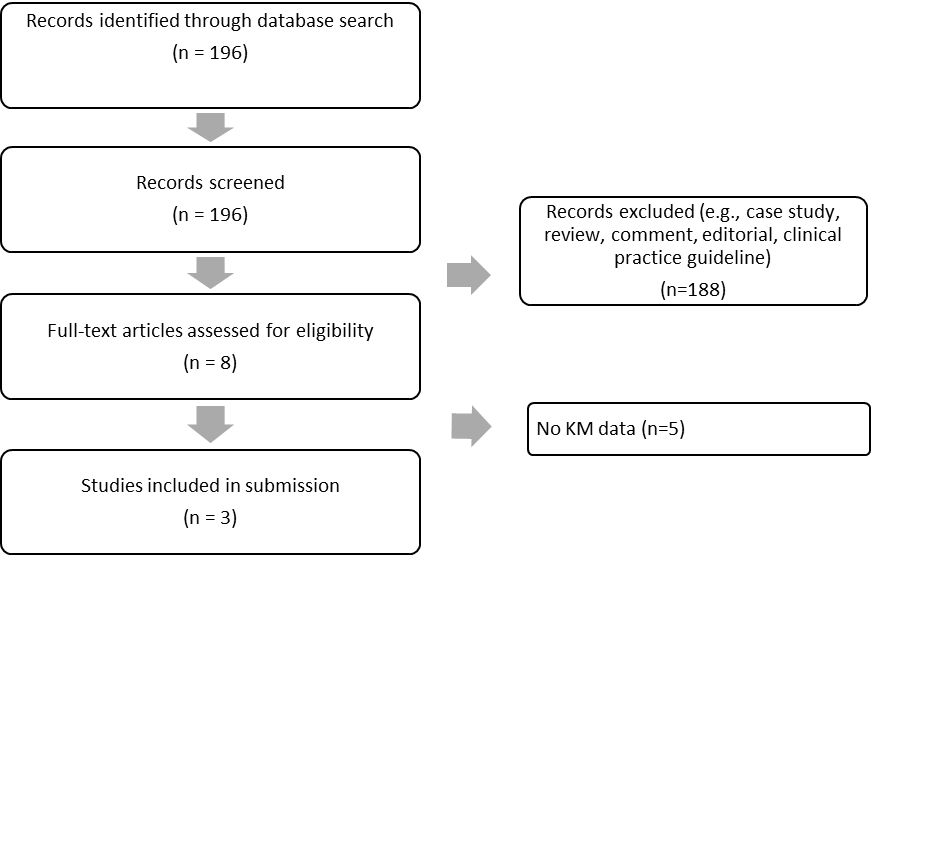 |

Fig. 1. PRISMA flow chart for ROS1 studies


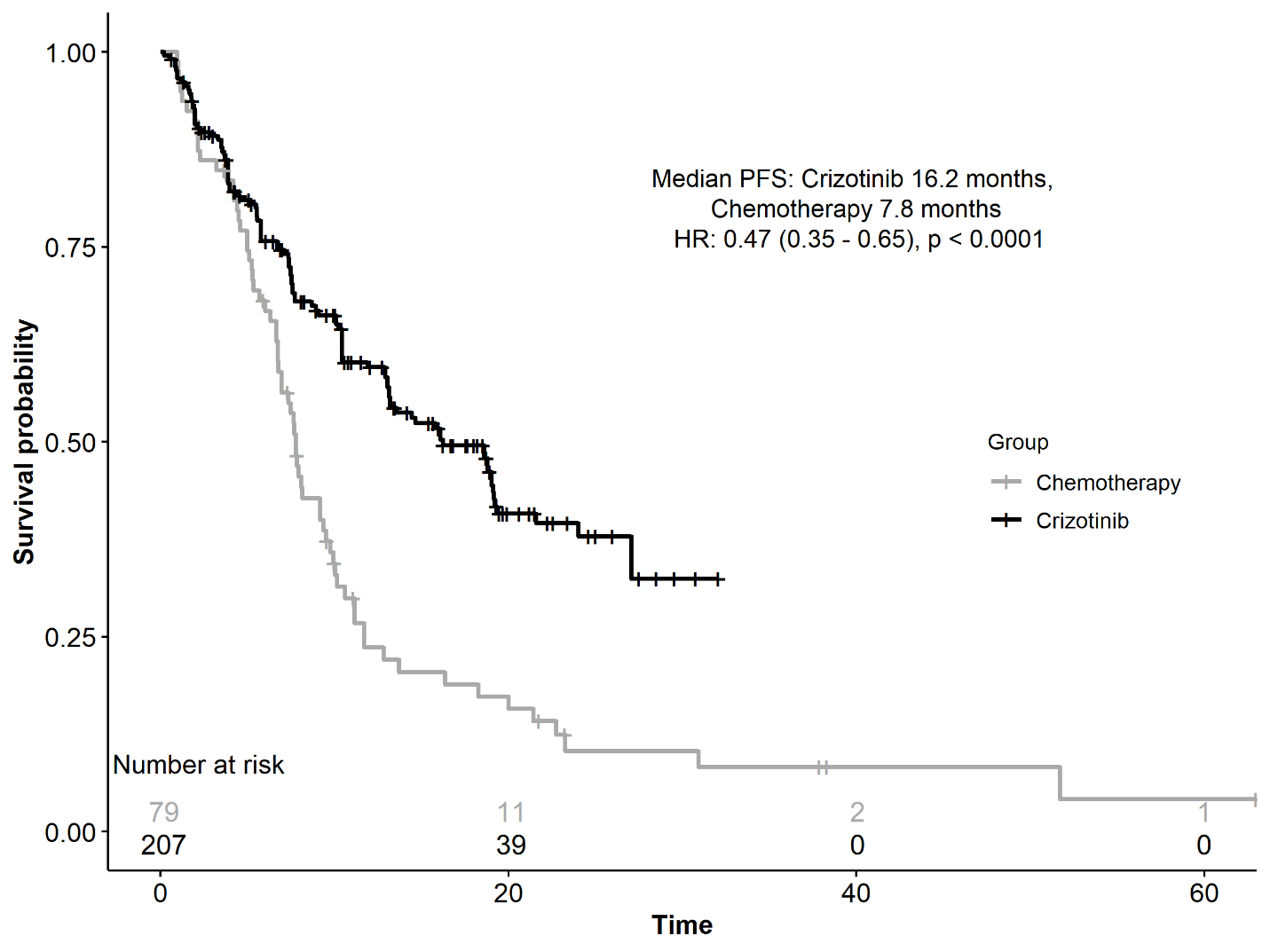


Fig. 2. Kaplan-Meier curve from combined analysis for progression-free survival (PFS) among ROS1+ NSCLC patients


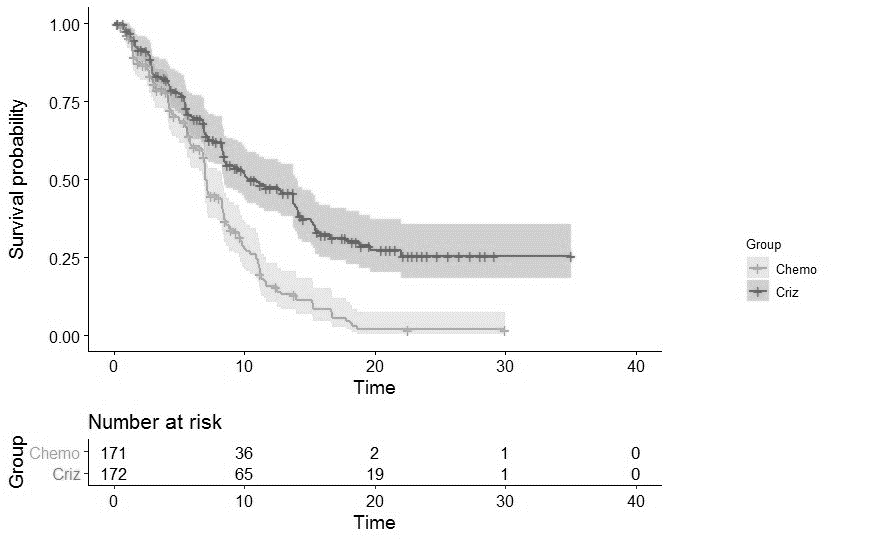


Fig. 3. Kaplan-Meier curve of PROFILE 1014 for progression-free survival (PFS) among ALK+ NSCLC patients for each treatment

Chemo = Chemotherapy; Criz = Crizotinib

Fig. 4. Kaplan-Meier curve and parametric curve of combined analysis for progression-free survival (PFS) for each treatment group with hazard ratio applied to patients undergoing maintenance pemetrexed in chemotherapy group

Chemo = Chemotherapy; Criz = Crizotinib

Fig. 5. Results from one-way deterministic sensitivity analysis

Chemo = Chemotherapy; Criz = Crizotinib; PFS = Progression-free survival; OS = Overall survival

References

1. Shaw AT, Ou S-HI, Bang Y-J, et al. Crizotinib in ROS1 -Rearranged Non–Small-Cell Lung Cancer. N Engl J Med. 2014;371(21):1963-1971. doi:10.1056/NEJMoa1406766

2. Wu Y-L, Yang JC-H, Kim D-W, et al. Phase II Study of Crizotinib in East Asian Patients With ROS1-Positive Advanced Non–Small-Cell Lung Cancer. J Clin Oncol. 2018:JCO.2017.75.558. doi:10.1200/JCO.2017.75.5587

3. Mazières J, Rouvière D, D.Milia J, et al. Crizotinib therapy for advanced lung adenocarcinoma and a ROS1 rearrangement: Results from the EUROS1 cohort. J Clin Oncol. 2015;33(9):992-999. doi:10.1200/JCO.2014.58.3302

4. Song Z, Su H, Zhang Y. Patients with ROS1 rearrangement-positive non-small-cell lung cancer benefit from pemetrexed-based chemotherapy. Cancer Med. 2016;5(10):2688-2693. doi:10.1002/cam4.809

5. Zhang L, Jiang T, Zhao C, et al. Efficacy of crizotinib and pemetrexed-based chemotherapy in Chinese NSCLC patients with &lt;i&gt;ROS1&lt;/i&gt; rearrangement. Oncotarget. 2016;7(46). doi:10.18632/oncotarget.12612

6. Drilon A, Bergagnini I, Delasos L, et al. Clinical outcomes with pemetrexed-based systemic therapies in RET-rearranged lung cancers. Ann Oncol. 2016;27(7):1286-1291. doi:10.1093/annonc/mdw163

7. Kim HR, Lim SM, Kim HJ, et al. The frequency and impact of ROS1 rearrangement on clinical outcomes in never smokers with lung adenocarcinoma. Ann Oncol. 2013;24(9):2364-2370. doi:10.1093/annonc/mdt220

8. Borghaei H, Brahmer JR, Paz-Ares L, et al. Nivolumab in Nonsquamous Non-Small-Cell Lung Cancer. N Engl J Med. 2016;374(5):493-494. doi:10.1056/NEJMc1514790

9. Brahmer JR, Reckamp KL, Baas P, Crino L, Eberhardt WEE. Nivolumab versus Docetaxel in Advanced Squamous-Cell Non-Small Cell Lung Cancer. N Engl J Med. 2015;373(2):123-135. doi:10.1056/NEJMoa1507643.Nivolumab

10. Herbst RS, Baas P, Kim DW, et al. Pembrolizumab versus docetaxel for previously treated, PD-L1-positive, advanced non-small-cell lung cancer (KEYNOTE-010): A randomised controlled trial. Lancet. 2016;387(10027):1540-1550. doi:10.1016/S0140-6736(15)01281-7

11. Vansteenkiste J, Fehrenbacher L, Spira AI, et al. Atezolizumab monotherapy vs docetaxel in 2L/3L non-small cell lung cancer: Primary analyses for efficacy, safety and predictive biomarkers from a randomized phase II study (POPLAR). Eur J Cancer. 2015;51:abstr 14LBA. doi:10.1016/S0959-8049(15)30072-1

12. Solomon BJ, Mok T, Kim D-W, et al. First-Line Crizotinib versus Chemotherapy in ALK -Positive Lung Cancer. N Engl J Med. 2014;371(23):2167-2177. doi:10.1056/NEJMoa1408440

13. Smit EF, Burgers SA, Biesma B, et al. Randomized phase II and pharmacogenetic study of pemetrexed compared with pemetrexed plus carboplatin in pretreated patients with advanced non-small-cell lung cancer. J Clin Oncol. 2009;27(12):2038-2045. doi:10.1200/JCO.2008.19.1650
